# A Deep Machine Learning-Based Assistive Decision System for Intelligent Load Allocation under Unknown Credit Status

**DOI:** 10.1155/2022/5932554

**Published:** 2022-09-08

**Authors:** Wenjing Yan, Hong Wang, Min Zuo, Haipeng Li, Qingchuan Zhang, Qiang Lu, Chuan Zhao, Shuo Wang

**Affiliations:** ^1^National Engineering Research Centre for Agri-product Quality Traceability, Beijing Technology and Business University, Beijing 100048, China; ^2^Capinfo Company Ltd., Beijing 100010, China; ^3^School of Mechanical Engineering, Beijing Institute of Technology, Beijing 100081, China

## Abstract

Nowadays, the banks are facing increasing business pressure in loan allocations, because more and more enterprises are applying for it and financial risk is becoming vaguer. To this end, it is expected to investigate effective autonomous loan allocation decision schemes that can provide guidance for banks. However, in many real-world scenarios, the credit status information of enterprises is unknown and needs to be inferred from business status. To handle such an issue, this paper proposes a two-stage loan allocation decision framework for enterprises with unknown credit status. And the proposal is named as TLAD-UC for short. For the first stage, the idea of deep machine learning is introduced to train a prediction model that can generate credit status prediction results for enterprises with unknown credit status. For the second stage, a dynamic planning model with both optimization objective and constraint conditions is established. Through such model, both the profit and risk of banks can be well described. Solving such a dynamic planning model via computer simulation programs, the optimal allocation schemes can be suggested.

## 1. Introduction

Since the rise of banks, loans have become the most common way of financing the process of enterprise development. Two main bodies are involved in general loan activities [1]. One is the communities that provide funds, such as financial companies and banks, while the other is the communities that apply for borrowing funds, such as enterprises [2, 3]. With the continuous growth of urbanization and modernization, the business volume of loans shows a gradually increasing trend [4]. This not only brings greater capital pressure to banks but also increases some uncertain financial risk for banks [5, 6]. Because the operation status and qualification of many enterprises are diverse, a great challenge is posed to the later management of banks [7, 8]. Therefore, how to calculate the optimal loan schemes that can maximize profit or minimize risk for banks under limited capital, serves as an important problem [9, 10].

It is never an easy task to determine the optimal loan allocation schemes for banks [11]. Although there are some research works focused on this issue, most of them did not consider the limitation of the total capital amount [12, 13]. They focused more on the scenes that decide whether to provide loans to specific enterprises [14–16]. They did not consider more substantive issues such as amount setting or global risk [17–19]. In addition, in the process of loan review, the most important consideration of enterprises is credit status. In the existing research works, they basically assume that the credit status of enterprises is known [20, 21]. However, in many actual business scenarios, the credit status of enterprises is unknown. The circumstances bring many challenges to the formulation of loan allocation plans. Therefore, how to generate the optimal loan allocation scheme for enterprises with unknown credit status is essentially a more realistic problem.

To deal with such an issue, this paper proposes a two-stage loan allocation decision framework for enterprises with unknown credit status, which is named as TLAD-UC for short. For the first stage, it is expected to tackle with the issue that credit status for enterprises is unknown. As a consequence, a typical machine learning named as K-nearest neighbor (KNN) is utilized here to predict credit status for enterprises. Specifically, a historical dataset that records information of 123 enterprises with credit status is selected to train the machine learning-based prediction models. And the trained models will be used to generate prediction results for enterprises with unknown credit status. For the second stage, a dynamic planning model is formulated to fit the decision process of banks, in which profit and risk are both expressed with quantified expressions. The dynamic planning model is composed of both optimization objective and constraint conditions. By solving the dynamic planning model, the optimal loan allocation decision schemes can be obtained. The main contributions of this paper can be summarized in three aspects.

1. It is recognized that loan allocation for enterprises with unknown credit status is challenging.
2. We propose TLAD-UC which is a two-stage loan allocation decision framework for enterprises with unknown credit status.
3. Simulation is conducted on a real-world dataset to demonstrate the workflow of the proposed TLAD-UC.

## 2. Preliminaries

**Figure 1:**
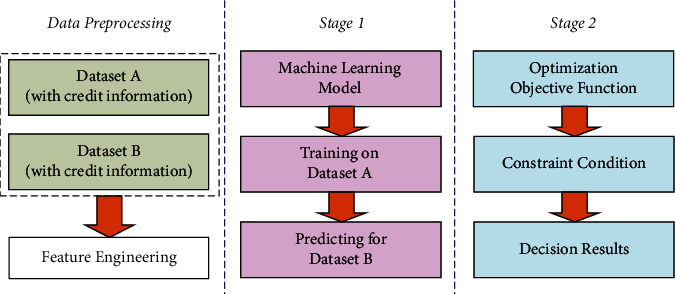
The main architecture of the proposed TLAD-UC.

Two datasets are involved in this work. Dataset A records some information of 123 enterprises and has credit status information for them. Dataset B only has some basic information of 302 enterprises yet has no credit status information. Inside both datasets, each enterprise has some business records of input invoices and sale invoices as their basic features. Let *n* denote the index number of enterprises that ranges from 1 to *N*, and the *N* equals to the number of enterprises in corresponding datasets. Taking the 123 enterprises with credit information as references, the main goal is to determine loan allocation schemes for the 302 enterprises without credit information. To handle such a problem, the TLAD-UC is implemented via two stages. As is shown in Figure 1, the two stages involved in the architecture of TLAD-UC are the machine learning stage and the optimization decision stage.

For the first stage, initial business data is preprocessed into a format that is suitable for data analysis models. And then, the KNN model is trained on the basis of dataset A. After training, it can directly predict unknown credit information for 302 enterprises in dataset B. For the second stage, the profit and risk of the 302 enterprises are quantified via mathematical expressions. On such a basis, a dynamic planning model with both optimization and constraint conditions is established for the side of banks. Then, the dynamic planning model can be solved by using computer simulation programs to search for optimal solutions for the planning model. Naturally, the optimal loan allocation schemes can be obtained after a solution to the optimization objective.

## 3. The Proposed Approach

### 3.1. Data Preprocessing

In the beginning, the initial datasets need some basic procedures to extract features. The following procedures are the basic process of feature engineering:

(1) For each enterprise, the total amount of its input invoices and the total amount of its sale invoices are respectively counted via aggregation of all related records that are labeled as “valid”. For the *n*-th enterprise, its amount of input invoices and amount of sale invoices are denoted as *F*_*n*,1_ and *F*_*n*,2_, separately.
(2) It is noted that some of the values in *F*_*n*,1_ and *F*_*n*,2_ are less than 0, which means that the corresponding business record is a chargeback record. Similarly, the total amount of chargeback for each enterprise is counted. For the *n*-th enterprise, its amount of chargeback amount in input invoices and sale invoices are denoted as *F*_*n*,3_ and *F*_*n*,4_, respectively.
(3) The ratio of chargeback data can be computed for both input invoices and the sale invoices via the following two formulas:
  (1) $$\begin{array}{c} F_{n,5}=\frac{F_{n,3}}{F_{n,1}},\\ F_{n,6}=\frac{F_{n,4}}{F_{n,2}}. \end{array}$$
For the *n*-th enterprise, its ratio of chargeback data in input invoices and sale invoices are denoted as *F*_*n*,5_ and *F*_*n*,6_, respectively.
(4) It is noted that values in *F*_*n*,1_ and *F*_*n*,2_ do not equal to the turnover amount because there exists the tax. The real turnover amount for a business record equals the sum of the invoice amount and tax amount. Thus, the turnover amount of input business and sale business can be calculated and denoted as *F*′_*n*,7_ and *F*′_*n*,8_, respectively. For the two indicators, their average values of one day can be computed as follows:
  (2) $$\begin{array}{c} F_{n,7}=\frac{{F^{\prime }}_{n,7}}{365},\\ F_{n,7}=\frac{F_{n,8}^{\prime }}{365}. \end{array}$$
For the *n*-th enterprise, its ratio of chargeback data in input invoices and sale invoices are denoted as *F*_*n*,7_ and *F*_*n*,8_, respectively.
(5) For each enterprise, the number of chargeback records is counted. For the *n*-th enterprise, such feature is denoted as *F*_*n*,9_.
(6) For each enterprise, its turnover amount needs to be processed by introducing logarithmic operations, which can be calculated as follows:
  (3) $$\begin{array}{c} F_{n,10}=\log_{2}\left(F_{n,1}\right)+\log_{2}\left({F^{\prime }}_{n,7}\right),\\ F_{n,11}=\log_{2}\left(F_{n,2}\right)+\log_{2}\left({F^{\prime }}_{n,8}\right). \end{array}$$
For the *n*-th enterprise, its two features are denoted as *F*_*n*,10_ and *F*_*n*,11_, respectively.
(7) For each enterprise, its turnover amount also needs to be processed by introducing logarithmic operations, which can be calculated as follows:
  (4) $$\begin{array}{c} F_{n,12}=\left|\log_{2}\left({F^{\prime }}_{n,7}\right)\right|,\\ F_{n,13}=\left|\log_{2}\left({F^{\prime }}_{n,8}\right)\right|. \end{array}$$
For the *n*-th enterprise, its two features are denoted as *F*_*n*,12_ and *F*_*n*,13_, respectively.
(8) For each enterprise, it has four possible label options which correspond to four credit ratings. The label of *n*-th enterprise is denoted as *y*_*n*_.

**Figure 2:**
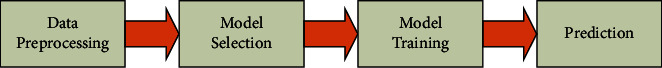
The main workflow of the machine learning algorithms.

As shown in Figure 2, the main workflow of machine learning algorithms is composed of four procedures: data preprocessing, model selection, model training, and prediction. Having finished the data preprocessing, it is expected to implement model selection and model training. For the *n*-th enterprise, its thirteen features can be denoted as *F*_*n*,*m*_, where *m* ranges from 1 to 13. Given *F*_*n*,*m*_, it is expected to generate prediction results for it. This process can be represented as the following formula:

(5) $$\begin{array}{c} F_{n,m}⟶y_{n}. \end{array}$$

To realize such a goal, the idea of machine learning is then introduced.

### 3.2. Prediction of Unknown Credit Information

As has been mentioned above, the dataset A has credit information and dataset B has no credit information. Thus, the dataset A is viewed as a golden dataset, from which unknown pattern rules can be discovered. Viewing the dataset A as a training set and the dataset B as the set to be predicted, a typical machine learning model named as KNN is selected here for this purpose.

The full name of KNN is K-nearest neighbors, and the KNN can be used for both classification problems and regression problems. The KNN realizes classification tasks or regression tasks by measuring the distance between different eigenvalues. Naturally, the selection of K-nearest neighbors is upon the basis of distance in sample spaces. The KNN is a quite easy but special machine learning algorithm, as it lacks the general learning process. Its working principle is to divide the feature vector space by using the training data and take the division results as the final algorithm model. After entering the unlabeled data, it is supposed to compare each feature of the unlabeled data with the corresponding feature of the data in the sample set. Then, the classification labels of the data with the closest features (nearest neighbors) in the sample are extracted.

**Figure 3:**
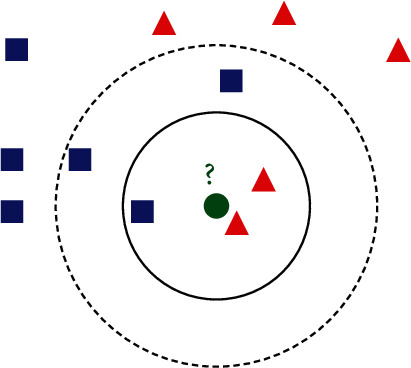
An example to illustrate the KNN algorithm.

We take Figure 3 as an example to illustrate the basic principles of KNN. Inside the figure, red points and blue points refer to samples that have been labeled. They belong to two different classes. It is expected to generate classification results for the green point. When *K* equals to 3, the selected neighbors for the green point include two red points and one blue point. According to the majority voting rule, the green point will be annotated as the class of red points. When *K* equals to 5, the selected neighbors for the green point include two red points and three blue points. According to the majority voting rule, the green point will be annotated as the class of blue points. From this example, it can be deduced that the setting of *K* is quite important in KNN because the constitution of neighboring samples may be diverse with different settings of *K*. Then, there is an essential problem in KNN, how to measure the distance in sample spaces?

In this work, the most prevalent distance measurement named as “Euclidean distance” is selected for use. Supposing that there are two sample points denoted as *α* and *β* in sample spaces, they are both four-dimensional samples. The Euclidean distance between *α* and *β* is calculated as the following formula:

(6) $$\begin{array}{c} \text{Dist}\left(\alpha ,\beta \right)=\sqrt{{\left(\alpha_{1}-\beta_{1}\right)}^{2}+{\left(\alpha_{2}-\beta_{2}\right)}^{2}+{\left(\alpha_{3}-\beta_{3}\right)}^{2}+{\left(\alpha_{4}-\beta_{4}\right)}^{2}}. \end{array}$$

It can be seen from the formula that the value of Dist(*α*, *β*) is sensitive to a diverse value range. For example, if the value range of *α*_1_ and *α*_2_ is larger than other features, the final value of Dist(*α*, *β*) will be influenced to some extent. To reduce such an effect, it is supposed to make normalization operations towards all the feature values. Universally, the value range of normalization is fixed as [0,1]. Taking *α*_1_ as an example, the normalization procedure can be calculated as follows:

(7) $$\begin{array}{c} \alpha_{1}^{\text{new}}=\frac{\alpha_{1}^{\text{old}}-\min\left(\alpha_{1}\right)}{\max\left(\alpha_{1}\right)-\min\left(\alpha_{1}\right)}, \end{array}$$

where min(*α*_1_) denotes the minimum value in all the *α*_1_ values in sample spaces, and max(*α*_1_) denotes the maximum value in all the *α*_1_ values in sample spaces. Naturally, all the features need to be normalized before substituting into models.

Therefore, major procedures of the KNN algorithm can be described as follows:

1. The distance between the test data and each training data is calculated.
2. All the possible neighbors are sorted by an increase in distance.
3. K samples with the nearest distance are selected as the neighbors.
4. The occurrence frequency of the category to which these *k* samples belong to is counted.
5. The category with the highest frequency in the K samples is returned as the prediction classification of the test data.

**Figure 4:**
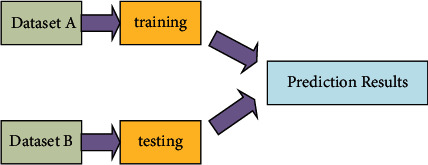
Workflow of the KNN model used in this work.

And the above process can be summarized in Figure 4.

### 3.3. Model Evaluation and Prediction

After training a KNN model, the credit status information in dataset B can be calculated accordingly. Before that, we would like to evaluate the performance of the KNN model. For dataset A, it is further divided into two parts the training part and the evaluation part. Of all the 123 samples, the training part has 93 samples, and the evaluation part has 30 samples. The 93 samples are used to train a KNN model and the 30 samples are used to evaluate the performance of the KNN model because the 30 samples have been labeled. Their labels are removed at first and then are compared with predicted labels.

**Figure 5:**
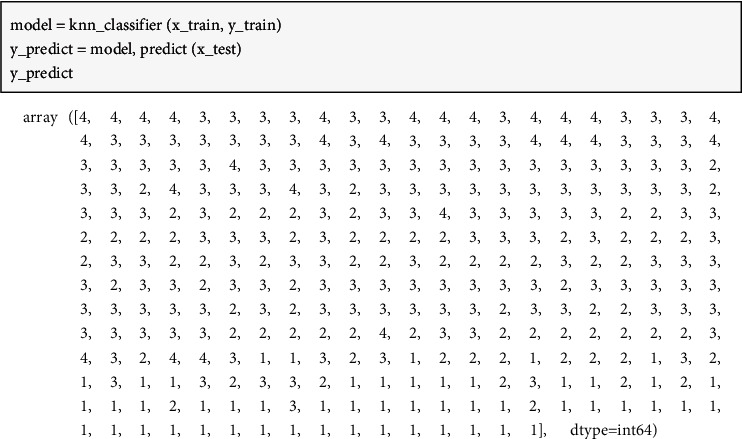
Running result of the KNN algorithm for prediction.

The KNN model outputs prediction results for the 30 results, of which 18 of them are correct and the other 12 of them are incorrect. Thus, we can say that prediction accuracy in the evaluation data is 0.6. Although such accuracy is still not ideal, it can have some guidance for enterprises with unknown credit status information. Because it can predict credit status information for the enterprises with some reliability. After training, the KNN model is implemented on dataset B to predict unknown credit status for them. Then KNN model is implemented on computers with the use of Python language. The running result of the computer program can be demonstrated in Figure 5.

In the next stage, the optimization decision model will be formulated on the basis of such prediction results. To sum up, 27 enterprises are labeled as credit rating A, 149 enterprises are labeled as credit rating B, 74 enterprises are labeled as credit rating C, and 52 enterprises are labeled as credit rating *D*.

### 3.4. Optimization Decision

To generate optimal allocation decisions for enterprises, a dynamic planning model is formulated in this section to realize this purpose.

From the side of banks, their total income from loan activities can be represented as the following formula:

(8) $$\begin{array}{c} I=c_{1}d_{1}s_{1}Q_{1}+c_{2}d_{2}s_{2}Q_{2}+c_{3}d_{3}s_{3}Q_{3}+c_{4}d_{4}s_{4}Q_{4}, \end{array}$$

*c*_1_, *c*_2_, *c*_3_, and *c*_4_ are the number of enterprises with four different credit ratings. *d*_1_, *d*_2_, *d*_3_, and *d*_4_ are loan amounts for enterprises with four different credit ratings. *s*_1_, *s*_2_, *s*_3_, and *s*_4_ are interest ratios for enterprises with four different credit ratings. *Q*_1_, *Q*_2_, *Q*_3_, and *Q*_4_ denote the proportion of no default for enterprises with four different credit ratings.

And for the side of banks, their risk in loan activities can be represented as the following formula:

(9) $$\begin{array}{c} R=c_{1}^{2}d_{1}^{2}s_{1}^{2}t_{1}\left(1-t_{1}\right)+c_{2}^{2}d_{2}^{2}s_{2}^{2}t_{2}\left(1-t_{2}\right),\\ +c_{3}^{2}d_{3}^{2}s_{3}^{2}t_{3}\left(1-t_{3}\right)+c_{4}^{2}d_{4}^{2}s_{4}^{2}t_{4}\left(1-t_{4}\right). \end{array}$$

Among, *t*_1_, *t*_2_, *t*_3_ and *t*_4_ denote the ratio of enterprises with four different credit ratings.

Besides, there are also some constraint conditions to be satisfied as follows:

1. *c*_1_*d*_1_+*c*_2_*d*_2_+*c*_3_*d*_3_+*c*_4_*d*_4_;
2. 0 ≤ *d*_1_, *d*_2_, *d*_3_, *d*_4_ ≤ *A*;
3. 0.04 ≤ *s*_*i*_ ≤ 0.15;
4. *s*_1_ ≤ *s*_2_ ≤ *s*_3_ ≤ *s*_4_.

Here, *A* denotes the total amount that can be used for loan activities in banks.

Further, the total profit for the side of banks can be represented as the following formula:

(10) $$\begin{array}{c} TP=c_{1}d_{1}s_{1}t_{1}\left(1-L_{1}\right)+c_{2}d_{2}s_{2}t_{2}\left(1-L_{2}\right),\\ +c_{3}d_{3}s_{3}t_{3}\left(1-L_{3}\right)+c_{4}d_{4}s_{4}t_{4}\left(1-L_{4}\right), \end{array}$$

among, *t*_1_, *t*_2_, *t*_3_ and *t*_4_ denote customer loss ratio of enterprises with four different credit ratings. Then, the optimization objective can be formulated from two aspects: risk minimization and profit maximization.

For risk minimization, the following optimization model can be formulated as follows:

(11) $$\begin{array}{c} \begin{array}{c} \min R\\ s.t.\left\{\begin{array}{c} c_{1}d_{1}+c_{2}d_{2}+c_{3}d_{3}+c_{4}d_{4}=A\\ 0\leq d_{1},d_{2},d_{3},d_{4}\leq A\\ 0.04\leq s_{i}\leq 0.15\\ TP\geq 0.07 \end{array}\right. \end{array}. \end{array}$$

Substituting *c*_1_, *c*_2_, *c*_3_, *c*_4_, *d*_1_, *d*_2_, *d*_3_, *d*_4_, *L*_1_, *L*_2_, and *L*_3_ into the model, the total profit and total risk can be written as follows:

(12) $$\begin{array}{c} \text{TP}=2.92d_{2}s_{2}+2.84d_{3}s_{3},\\ R=2.92d_{2}^{2}s_{2}^{2}+2.84d_{3}^{2}s_{3}^{2}. \end{array}$$

Assuming that the total amount for loan activities is set as 1, the optimal allocation scheme is computed as follows:

(13) $$\begin{array}{c} d_{1}=0.72,d_{2}=0.18,d_{3}=0.10. \end{array}$$

It is noted that enterprises with credit rating D will not be approved for loans here. And the interest ratio is set at 0.08.

For profit maximization, the following optimization model can be formulated as follows:

(14) $$\begin{array}{c} \begin{array}{c} \min TP\\ s.t.\left\{\begin{array}{c} c_{1}d_{1}+c_{2}d_{2}+c_{3}d_{3}+c_{4}d_{4}=A\\ 0\leq d_{1},d_{2},d_{3},d_{4}\leq A\\ 0.04\leq s_{i}\leq 0.15\\ R\leq 0.03 \end{array}\right. \end{array}. \end{array}$$

Substituting *c*_1_, *c*_2_, *c*_3_, *c*_4_, *d*_1_, *d*_2_, *d*_3_, *d*_4_, *L*_1_, *L*_2_, and *L*_3_ into the model, the optimal decision for allocation schemes can be represented as follows:

(15) $$\begin{array}{c} d_{1}=0.48,d_{2}=0.28,d_{3}=0.24. \end{array}$$

It is noted that enterprises with credit rating D will not be approved for loans here. And the interest ratio is set at 0.15.

**Figure 6:**
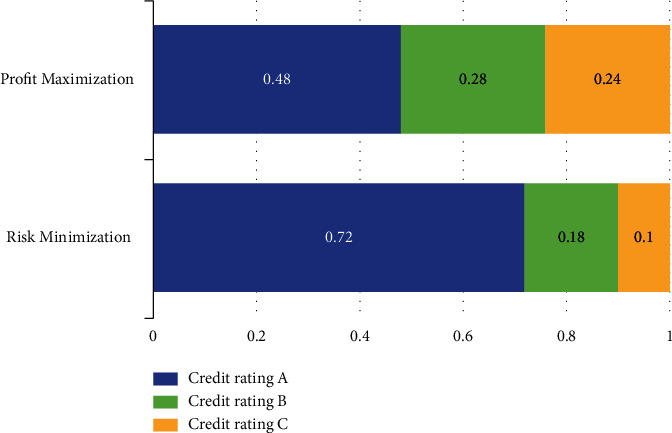
Final allocation results for the two situations.

**Figure 7:**
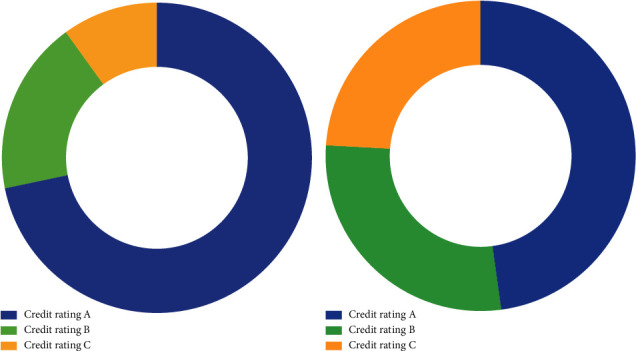
The suggested allocation schemes are under two different situations. (a) A scheme under risk minimization. (b) A scheme under profit maximization.

**Figure 8:**
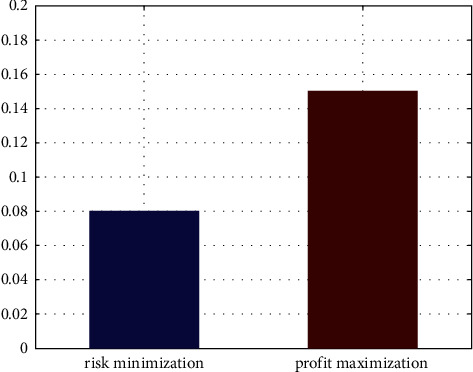
Final interest rate results for the two situations.

In order to visualize the allocation results more clearly, Figure 6 demonstrates the allocation results of three kinds of enterprises via a stacked bar chart. Inside the figure, the blue bar corresponds to allocation results for enterprises with credit rating A, the green bar corresponds to allocation results for enterprises with credit rating B, and the yellow bar corresponds to allocation results for enterprises with credit rating C, while no allocation is provided for enterprises with credit rating D. And the results under two situations are also illustrated respectively in Figure 7, in which two subfigures correspond to situations of risk minimization and profit maximization. We also make a visualization of interest rate under two situations in Figure 8. It is a bar chart with two main bars, in which the blue bar corresponds to the interest rate under risk minimization and the red bar corresponds to the interest rate under profit maximization.

## 4. Discussion about Machine Learning Application

This work deals with loan allocation decision situations where the credit status information of enterprises is unknown. As a consequence, this work introduces machine learning to predict unknown credit status information. The machine learning models are with simple principles and are more resilient compared with general mathematical modeling thoughts. Besides, there are many support services for the machine learning models, as many available interfaces can be directly imported. It can really act as an alternative for time-consuming manual decision tasks and can even be comparable to expert experience in some situations.

However, the machine learning models also have some limitations. The most common issue for machine learning models lies in the fact that they are highly reliable on labels and sample amounts, because the machine learning models need to be trained on the basis of gold labels in the training set and are quite sensitive to sample amount. In other words, there needs some cost to train an effective machine learning model. In addition, the selection of features may also have some effect on the fitting efficiency of machine learning models, which is attributed to the explainability problem of general machine learning models. Due to the weak explainability, the establishment of models may lead to many redundant labors. But on the whole, the machine learning models can still work as a feasible solution in many business scenarios.

## 5. Conclusion

This paper focuses on a smart finance task using machine learning methods. To complete unknown credit status information of users, this work uses the KNN model for this purpose. After that, a dynamic planning model is utilized to realize decision-making processes. The whole technical framework is named as TLAD-UC for short which is composed of two stages. A real-world dataset is selected to evaluate the performance of the proposed TLAD-UC. A case study is presented to display the workflow of the proposal. It is also noted that the current technique is still in the initial exploration of this area, and efficiency needs to be further improved in future works. Therefore, it is expected to improve technical methods and promote decision effect. And the idea of an autonomous decision may be considered in future works.

## Data Availability

The research data can be requested from the first author via e-mail.
